# Corrosion Protection of Q235 Steel Using Epoxy Coatings Loaded with Calcium Carbonate Microparticles Modified by Sodium Lignosulfonate in Simulated Concrete Pore Solutions

**DOI:** 10.3390/ma14081982

**Published:** 2021-04-15

**Authors:** Weilin Liu, Jiansan Li, Xiangqi Huang, Jinye Bi

**Affiliations:** School of Mechanical and Automotive Engineering, South China University of Technology, Guangzhou 510640, China; 201921003345@mail.scut.edu.cn (X.H.); 201921003388@mail.scut.edu.cn (J.B.)

**Keywords:** calcium carbonate microparticles, sodium lignosulfonate, Q235 steel, epoxy coating, corrosion inhibition, simulated concrete pore solution

## Abstract

In this study, calcium carbonate (CaCO_3_) microparticles having pH-sensitive properties were loaded with sodium lignosulfonate (SLS), a corrosion inhibitor. Scanning electron microscope (SEM), UV–VIS spectrophotometer (UV-vis), X-ray diffraction (XRD), and attenuated total reflection-Fourier-transform infrared spectroscopy (ATR-FTIR) were applied to evaluate the properties of the synthetic microparticles. This material could lead to the release of corrosion inhibitor under different pH conditions of the aqueous media. However, the extent of release of the corrosion inhibitor in the acidic media was higher, leading to enhanced shielding effect of the Q235 steel. These microparticles can serve as anti-corrosion additive for epoxy resin-coated Q235 steel. Electrochemical experiments were used to assess the anti-corrosive ability of the epoxy coatings in simulated concrete pore (SCP) solution, confirming the superior corrosion inhibition of the epoxy coating via incorporation of 5 wt % calcium carbonate microparticles loaded with SLS (SLS/CaCO_3_). The physical properties of coating specimens were characterized by water absorption, contact angle, adhesion, and pencil hardness mechanical tests.

## 1. Introduction

Among the various building materials used in civil engineering, concrete is considered as one of the most critical materials. Life span of the concrete mainly depends on the steel bars along with different other properties of concrete. Steel corrosion is one of the key factors reducing the life span of the concrete [1]. The corrosion initiation of reinforced concrete can be triggered by carbonation and chloride erosion [2,3], especially under severe environment including marine and saline lake environment. Under these environments, the protective layer on the steel bars gets quickly destroyed, which subsequently leads to the destruction of concrete and thereby damaging the reinforcement [4,5].

Therefore, efficient prevention and repair of the corrosion damage of the reinforced concrete structures are necessary. Various methods have been proposed for mitigating the corrosion damage including admixing of suitable corrosion inhibitors [6,7,8], selection of corrosion-resistant steel bars (such as galvanized steel bars and epoxy-coated steel bars) [9,10], and using electrochemical protection [11]. In terms of economic and technical point of view, a durable and well-bonded coating on steel can serve as a physical barrier against the ingression of potentially dangerous environmental corrosive agents. The coating allows the steel surface to tolerate corrosive ions from the external medium by providing a physical barrier [12,13,14]. The key characteristics of the protective level provided by coating are the adhesiveness and continuity of the coating applied on the metal surface and its reactivity with the environmental medium.

Epoxy resins have extensive applications in the protection of metallic corrosion due to their low costs and excellent anti-corrosive ability. The matrix of the metal is prevented from getting destroyed by the corrosive ions by the coating. However, most of the coatings are vulnerable against mechanical damage. Therefore, some researchers mixed corrosion inhibitors to the coating for enhancing the effective corrosion inhibition and mechanical properties of the coating [15,16]. However, if added directly to the coating, the corrosion inhibitors show certain reactivity towards certain components of the coating, which subsequently affect the mechanical strength and stability of the coating and simultaneously lead to environmental problems [11,17,18,19]. In the recent years, many researchers have applied carrier for the loading of inhibitors in order to prevent the reaction between the inhibitors and the coating, and thereby achieving optimized shielding properties of the coating [19].

Calcium carbonate (CaCO_3_) can be divided into three kinds of crystal polymorphs according to the crystalline form having similar composition but different structures, i.e., calcite, aragonite, and vaterite [20]. Through the series gradual decrease in thermal stability takes place with sequential increase in energy [21]. In the biological systems, CaCO_3_ is mostly present as calcite or aragonite. The thermodynamics of vaterite is unstable and quick transforms vaterite into calcite or aragonite in an aqueous solution. Vaterite is rare in the nature. In recent times, CaCO_3_ microspheres have received great attention thanks to their better mechanical and thermal stability, biocompatibility, and biodegradability [22,23,24,25]. Nano-particulate CaCO_3_ can be transformed into various structures and morphologies using modifiers, and it is possible to easily load them with the corrosion inhibitors [26,27]. CaCO_3_ powders have been used as filler in coating and as a carrier material to control the release rate [28]. This behavior does not lead to incompatibility between the particles and the coating. If CaCO_3_ microspheres loaded with corrosion inhibitors are embedded in the coating, slight modification of the coating is required and the modification step is a simple one.

Earlier reports in the literature have shown the application of epoxy coatings modified by CaCO_3_ microspheres with a variety of inhibitors over aluminum alloy as anti-corrosive coating [19]. These coatings were found to enhance the impact resistance, thermal stability, and corrosion resistance. However, with the increasing of added microspheres, tensile characteristic of the thin layer is reduced, leading to brittleness of the coating. Recent work demonstrated that CaCO_3_ microbeads have been modified with various inhibitors: cerium nitrate, salicylaldoxime, and 2,5-dimercapto-1,3,4-thiadiazolate to extend the corrosion inhibition ability of coating and impart pH sensitivity. It indicated that CaCO_3_ microbeads with cerium nitrate revealed good dispersibility, imparted pH sensitivity into epoxy coatings, and prolonged corrosion in AA2024 substrates [19]. Roma Raj et al. also investigated that CaCO_3_ microbeads worked as containers for a mixture of two inhibitors triethanolamine (TEA) and polyethylenimine (PEI) in epoxy coating and found that the coating pore resistance of the modified coating presented about four order of magnitude higher compared to the neat epoxy coating [29].

Sodium lignosulfonate (SLS) is a natural polymer and is being used as an anionic surfactant [30]. As a by-product from the production of pulp, SLS is harmless to the environment. SLS is soluble in water and in low concentration exists as a single molecule with a diameter of about 14–22 nm. With increasing concentration of SLS, SLS polymerizes and forms spherical particles with the diameter of about 0.5–1 µm. SLS has been reported to provide corrosion protection for steel. Ouyang et al. investigated the corrosion inhibition performances of carbon steel under recirculatory cooling water using SLS as an environmentally benign corrosion inhibitor [31]. SLS showed active inhibition properties on zinc in hydrochloric acid (HCl) aqueous solution [32].

This study explores the anti-corrosion properties of a novel composite prepared by the combination of SLS and pH-sensitive CaCO_3_ particles in the epoxy coatings applied on Q235 steel. Various physicochemical analytical methods were used to characterize the microspheres with or without SLS loading. After immersion in the SCP solutions containing 3.5 wt % sodium chloride, the inhibition protection of the coated Q235 coupons was evaluated by electrochemical measurements and physical properties tests. 

## 2. Experimental Procedures

### 2.1. Synthesis of SLS/CaCO_3_

CaCO_3_ microparticles were synthesized via the co-precipitation method. In the first step, 0.5 M CaCl_2_ (50 mL) (purchased from Macklin (Shanghai, China), ≥99.9%) was poured rapidly into an equal volume of 0.5 M Na_2_CO_3_ (purchased from Macklin, ≥99.9%) under vigorous stirring (40 °C, 60 s). The produced suspension was evacuated applying vacuum filtration with filter paper of 0.22 μm pore size and rinsed with deionized water and ethanol. Then, the synthesized microparticles were dried for 24 h at 50 °C. Dried calcium carbonate particles were soaked into a 15 wt% SLS solution (50 mL) (purchased from Macklin) in vacuum for 1 day. The suspension was separated using circulating water vacuum pump, followed by washing with Millipore ultrapure water and subsequent drying in an oven at 50 °C.

### 2.2. Preparation of the Epoxy Coating

The Epoxy resin (E44) and polyamide hardener (650) were provided by Zhenjiang Danbao Resin Co., Ltd. (Zhenjiang, China). Epoxy resin with CaCO_3_ microparticles with or without SLS loading was applied onto the prepared specimens. CaCO_3_ particles with SLS (3 wt% and 5 wt% of the gross weight of the hardener and epoxy resin) were added into ethanol under ultrasonic dispersing for 1 h. After that, the dispersion of CaCO_3_ particles with or without SLS loading was stirred with the epoxy resin for 10 min. Subsequently, the mixture of the hardener and epoxy resin keeping a weight ratio of 1:1 was stirred for 15 min before applying the coating mixture. Then, various coating formulations were applied on steel bar by dipping coating with the withdrawal rate of 18 cm/min. Finally, the coated steels were cured for 7 days.

### 2.3. Materials and Sample Preparation

**Figure 1:**
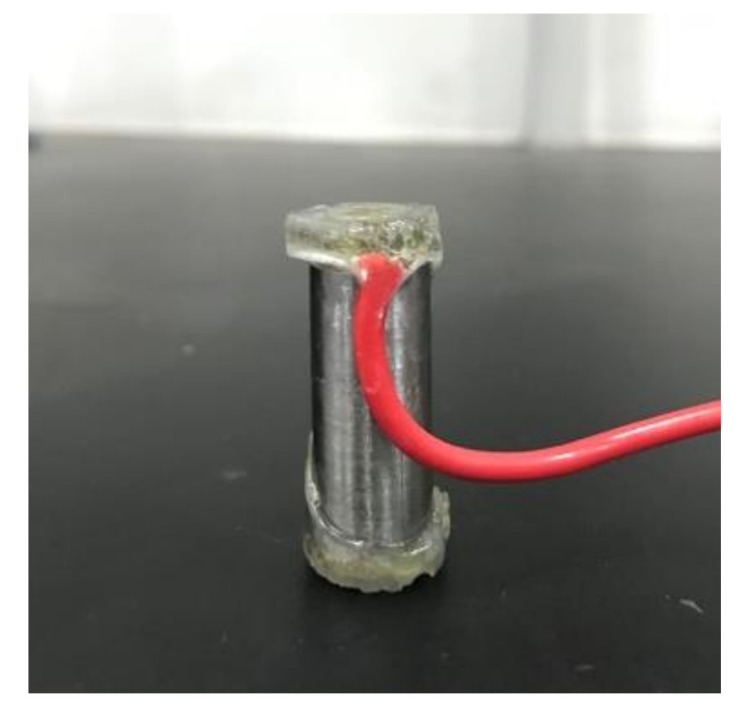
Coating steel bar.

Before coating, the Q235 carbon steel specimen (Dongguan Tengwei Metallic Material Co., Ltd., Dongguan, China) was abraded with metallographic paper (#400, #500, #600, #800 and #1000 garde), then sonicated in acetone for 5 min, followed by the subsequent rinsing with ethanol and deionized water [33]. The epoxy coatings were deposited onto the carbon steel bar measuring 12-mm diameter and 30-mm length. The picture of coating steel bar is shown in Figure 1. The chemical composition (wt%) of the carbon steel was 0.002 S, 0.003 N, 0.021 P, 0.021 O 0.13 C, 0.17 Si, 0.57 Mn, and balance Fe. Except the working surface, all the parts of the steel bar were sealed by the epoxy resin. The coated Q235 steel specimens were immersed in SCP solution at pH ~ 12.5 to evaluate their protective properties by means of electrochemical techniques.

## 3. Characterization of the Particles and Coated Samples

### 3.1. Scanning Electron Microscopy

SEM (Zeiss, Merlin, Jena, Germany) was applied to observe the micrographs of the blank and SLS-loaded microspheres (accelerating voltage of 5 kV). Appropriate amount of CaCO_3_ microbeads were stuck with conductive glue on the aluminum block, gilded, and then put into the sample chamber. In addition, SEM presented the fracture surface morphologies of the coatings after broken in liquid nitrogen.

### 3.2. X-ray Diffraction

X-Ray diffraction (XRD) study (Bruker, D8 Advance, Karlsruhe, Germany) using Cu-Kα radiation source was carried out to confirm the phase identification of CaCO_3_ particles before and after the loading with SLS. The data was collected between the angular range of 10° ≤ 2θ ≤ 80° employing a scanning rate of 2°/min with a 2θ step of 0.013°.

### 3.3. ATR-FTIR

The attenuated total reflection-Fourier-transformed infrared spectroscopy (Bruker, Vertex 70, Karlsruhe, Germany) was employed to analyze the structure of the CaCO_3_ microparticles employing wavelength range scanning from 4000 to 400 cm^−1^.

### 3.4. UV–Vis Spectrum 

UV–VIS spectrophotometer (YOKE INSTRUMENT, UV755B, Shanghai, China) was employed to measure the concentration of the released inhibitor in the aqueous solution from SLS/CaCO_3_ particles. The absorption peak at about 280 nm belongs to the benzene ring in the sodium lignosulfonate. The SLS concentration and its absorbance peak at 280 nm show good linear correlation. Standard curve was obtained by measuring the absorbance of known concentration of SLS in the aqueous solution and a linear plot was obtained. The loaded microspheres were dispersed in aqueous solution with varying pHs (4.0, 7.0, and 10.0) after different immersion times (24, 48, and 72 h).

### 3.5. Electrochemical Experiments

The steel was immersed in SCP test solutions for 15 days at ambient temperature. After stabilizing the open-circuit potential (OCP) for at least 30 min, inhibition properties of the coated-Q235 steel were measured using electrochemical impedance spectroscopy (EIS) spectra within the frequency range of 100 kHz to 10 mHz and oscillation amplitude of 20 mV from peak to peak utilizing Princeton 263A electrochemical workstation. Potentiodynamic polarization measurements also applied in the electrochemical workstation. The experimental potential range was in the range of −1.0 to +1.0 V (vs. OCP) and the scan rate was 0.8 mV/s. Reproducibility of the results was confirmed by carrying out each experiment at least thrice.

### 3.6. Water Absorption Test

Water absorption tests were used according to the ASTM D570 2005 standard in order to characterize the anti-corrosive ability of the coating. The weight of each coated samples was recorded before and after the immersion in deionized water at room temperature and was indicated as m_0_ and m_t_, respectively. Prior to weighing, the coating specimens were wiped with filtration paper. The coated samples were weighed after various immersion periods. Then, the water absorption Q_t_ was calculated by Equation (1) [34]

(1) $$Q_{t}=(m_{t}-m_{0})/m_{0}.$$

### 3.7. Contact Angle Tests

The contact angle estimations (DataPhysics, OCA40 Micro, Stuttgart, Germany) was carried out to analyze the wettability of the coating. Prior to the measurement, 3 μL distilled water was dropped on the surface of the coating steel. Each sample was measured three times.

### 3.8. Adhesion Measurements

The adhesion property of the coating is an effective criterion to evaluate the inhibition performance of the coatings. Adhesion test was performed according to ISO-2409: 2013 Standard. A coating surface was drawn out 100 grids and swept the surface with a brush. Then, a transparent adhesive tape was firmly stuck on the scratched surface and tore off with an angle of about 60° within 0.5–1.0 s. The spalling area of the coating was observed by magnifying glass. The adhesion level of the coating without the detached area can be classified as level 0, whereas coating surface with the lowest adhesion belong to level 5 [35].

### 3.9. Pencil Hardness Mechanical Test

The pencil hardness test was performed on the coatings according to ASTM D3363. The hardness of the wood pencils scale ranges from 6B to 6H. The test started from the pencil with minimum hardness (6B). Until the pencil scratched or dug the coating, the coating hardness was equated to the previous pencil. Gouge hardness and scratch hardness determined the hardness value. The first one presents the resistance of the coating to being cut, the second one indicates the mechanical resistance of the coating to being scratch [36].

## 4. Result and Discussion

### 4.1. Characterization of the Calcium Carbonate Particles

**Figure 2:**
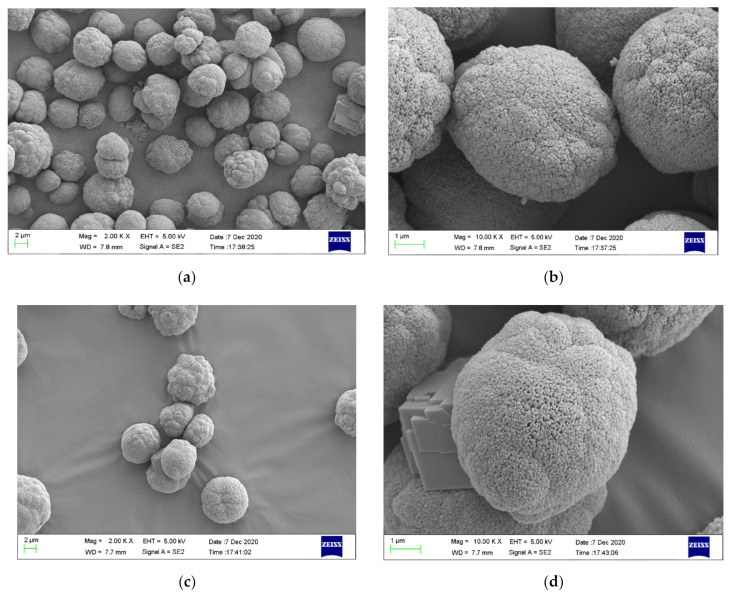
SEM images of (**a**) CaCO_3_ microparticles, (**b**) CaCO_3_ microparticles at higher magnification, (**c**) CaCO_3_ microparticles loaded with sodium lignosulfonate (SLS), and (**d**) CaCO_3_ microparticles loaded with SLS at higher magnification.

The morphological structures of the CaCO_3_ microbeads with or without SLS loading were investigated by SEM (Figure 2). As shown in Figure 2a, the morphological features of the CaCO_3_ microbeads prior to the SLS loading shows roughly spherical and oblong in shape. As confirmed by the magnified micrograph (Figure 2b), the CaCO_3_ microparticles are typical of porous vaterite and agglomerate to form larger microparticles which are composed of many small and nearly spherical microparticles. Each one small spherical microparticles will increase the surface area of CaCO_3_ microbeads and result in the generation of porous surface. Furthermore, CaCO_3_ microbeads without SLS loading are also composed of a small amount of prismatic calcite. These structures are conducive for the loading of the inhibitor. The average size of the unmodified CaCO_3_ microparticles is found to be around 2–10 μm. After the loading of SLS on the CaCO_3_ microparticles (Figure 2c,d), the original spherical morphology and diameter of the micro-agglomerates are maintained along with the observation of few prismatic calcite.

**Figure 3:**
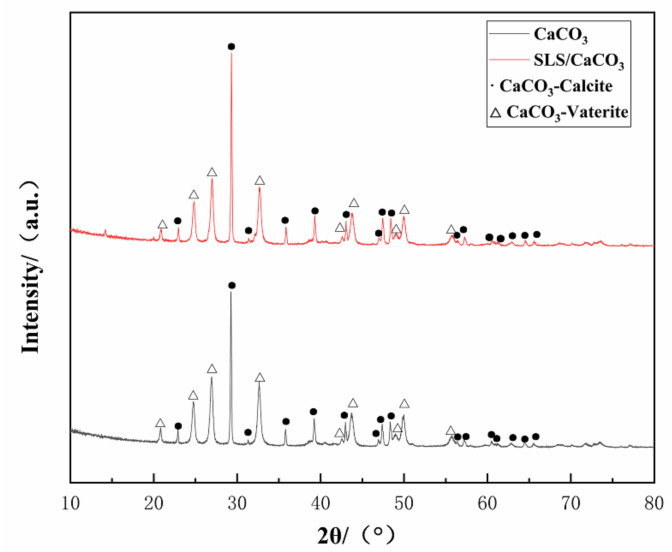
XRD spectrum of loaded and unloaded CaCO_3_ microparticles.

Crystalline structure of the synthesized CaCO_3_ (Figure 3) demonstrated characteristic diffraction peaks related to vaterite (JCPDS #72-0506) or calcite (JCPDS #99-0022), thus revealing the presence of mixed crystals of spherical vaterite and calcite. The high crystallinity of the obtained CaCO_3_ particles was confirmed from the pronounced peaks observed in the XRD pattern. Sixteen diffraction peaks in the XRD patterns at 2θ = 22.944, 29.311, 31.409, 35.849, 39.314, 43.045, 47.034, 47.455, 48.414, 56.463, 57.275, 60.535, 61.310, 62.909, 64.525, and 65.616° correspond to the respective planes of (012), (104), (006), (110), (113), (202), (024), (018), (116), (211), (122), (214), (119), (125), (300), (0012) of vaterite and the peaks at 2θ = 20.880, 24.883, 26.973, 32.711, 42.613, 43.767, 49.098, 49.946, and 55.768° correspond to the respective planes of (002), (100), (101), (102), (004), (110), (112), (104), and (202) for calcite. We estimated that the vaterite and calcite relative mole fractions in the CaCO_3_ microparticles can be correlated to the X-ray diffraction intensity from the following expression [37]:

(2) $$X_{v}=\frac{\;\left(I_{002v}+I_{100v}+\cdots I_{202v}\right)}{\left(I_{002v}+I_{100v}+\cdots I_{202v})+(I_{012c}+I_{104c}+\cdots +I_{0012c}\right)}$$

(3) $$X_{c}=1-X_{v},$$

where, X*_C_* and  X*_V_* are the calcite and vaterite mole fractions, respectively, *I_C_* is the X-ray diffraction intensity of the corresponding planes of calcite, and I_V_ is the X-ray diffraction intensity of planes of vaterite. 

The mole fractions of vaterite and calcite for the CaCO_3_ microparticles without SLS were found to be ~60.64% and 39.36%, respectively, while the corresponding values for the SLS-loaded CaCO_3_ microparticles were 55.95% and 44.05%, respectively.

Increasing pH of the synthetic solution due to the presence of SLS leads to the phase transformation of the synthesized CaCO_3_ microparticles from the vaterite to calcite. The dissolution of vaterite would lead to quick transformation of vaterite to calcite, so the addition of the SLS induced increasing rate of dissolution rate of the vaterite [38]. 

**Figure 4:**
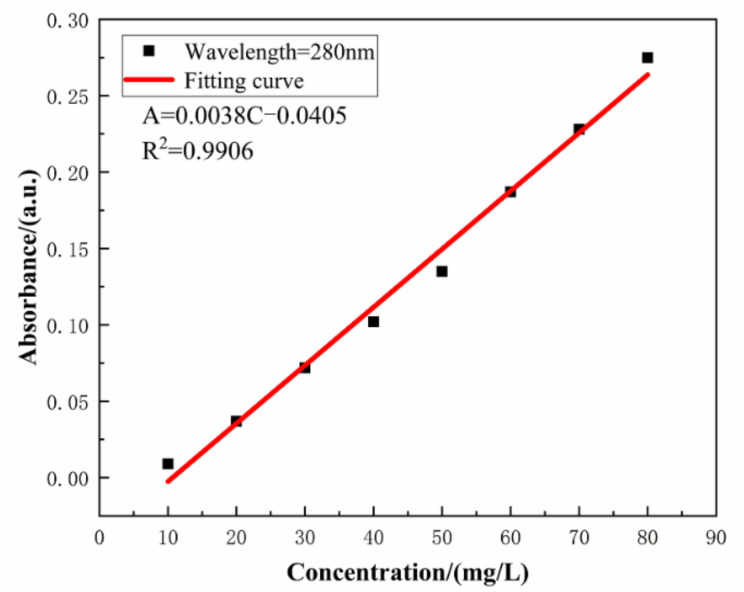
The concentration dependent absorbance of SLS in aqueous solution.

**Figure 5:**
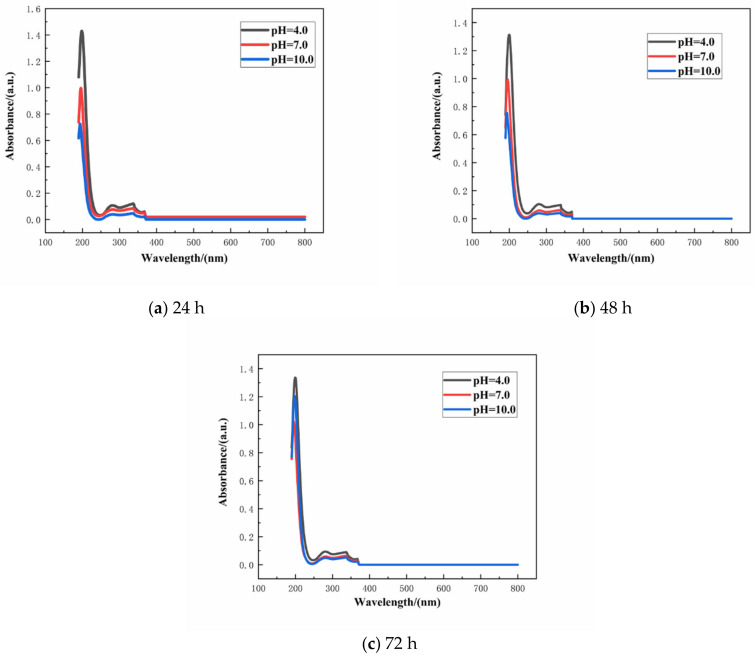
Release curves of SLS from the CaCO_3_ micro-containers at different pH values: (**a**) 24 h, (**b**) 48 h, and (**c**) 72 h.

The absorbance of the aqueous solution with varying concentration of SLS was monitored by UV–VIS spectrophotometer. Linearity of the standard plot with eight different concentrations of SLS (10, 20, 30, 40, 50, 60, 70, and 80 mg/L) is depicted in Figure 4 [39]. The standard curve can be used to calculate the concentration of SLS in aqueous solution. The structure of SLS contains functional groups capable of absorbing UV light and among them the main peak at 280 nm is due to the presence of benzene ring. The wavelength was scanned from 800 to 190 nm for monitoring the absorbance of SLS at 280 nm [40]. Standard concentration-absorbance equations of SLS were established within the concentration range of 10–80 mg/L (A = 0.0038 × C − 0.0405, R^2^ = 0.9906 where A, C and R^2^ are the absorbance, the concentration of SLS and the correlation coefficient, respectively). Under different pHs of the aqueous solution, the release of SLS/CaCO_3_ micro-containers were also studied employing UV–vis spectrophotometer. Figure 5 shows the time dependent change of the absorbance values of SLS from the CaCO_3_ micro-containers under various pH of the aqueous solution over 72 h (pH = 4.0, 7.0, and 10.0). These solutions were prepared with deionized water, 0.1 M acetic acid, and 0.1 M sodium hydroxides (NaOH). Table 1 lists the absorbance and concentration of the SLS released from the CaCO_3_ micro-containers for 72 h of immersion in various pH solutions. At all immersion times, the highest absorbance intensity was presented at pH 4. The released SLS concentration decreased with increasing pH values. In acidic and neutral media, the SLS released from the CaCO_3_ micro-containers rapidly in early time. However, in alkaline media, the concentration of SLS increased slowly as the immersion time increases. Release behavior of SLS from the CaCO_3_ under various pH of the aqueous solution showed higher release in the acidic medium in comparison to the alkaline medium. Therefore, the SLS-loaded CaCO_3_ micro-containers have promising applications in the reinforced concrete.

**Figure 6:**
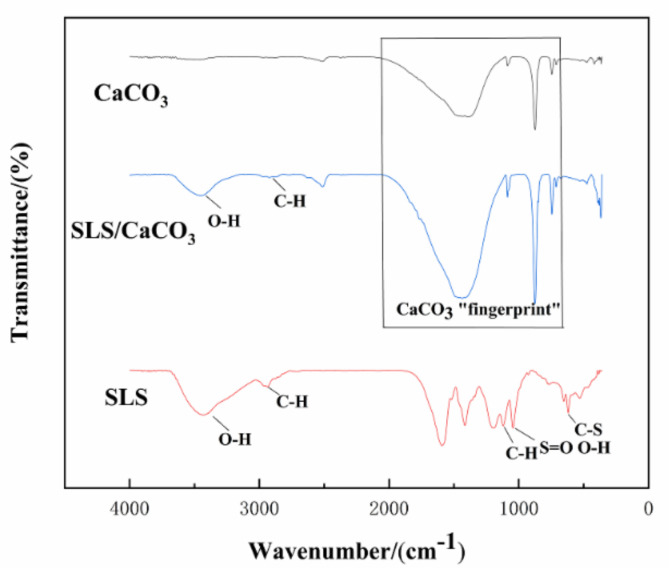
Attenuated total reflection-Fourier-transform infrared spectroscopy (ATR-FTIR) spectra of CaCO_3_, SLS, and SLS-modified CaCO_3._

Further details regarding the chemical composition of the CaCO_3_ micro-containers loaded with SLS were obtained by ATR-FTIR analysis. The absorption peaks at 713 and 876 cm^−1^ shown in Figure 6 can be assigned to the calcite. Two major bands at 745 and 1086 cm^−1^ are originated from the presence of vaterite. The broad areas of absorption in 1455–1490 cm^−1^ correspond to the amorphous calcium carbonate. The typical peaks attributed to calcite and vaterite remained unchanged in the spectra for the CaCO_3_ particles in absence or in presence of SLS. The presence of two weak peaks at 874–877 and 743–746 cm^−1^ corresponding to the out-of-plane and in-plane stretching of carbonate ions, respectively, confirm the presence of CaCO_3_. The carbonate ion is confirmed by the asymmetric stretching at 1446 cm^−1^ and the symmetric stretching at 1086 cm^−1^. The structure of SLS was also investigated by ATR-FTIR (Figure 6). The absorption peak at 2944 cm^−1^ is the signal of the C-H stretching for the methylene or methyl groups, and the characteristics spectral peak is detected at 618 cm^−1^ (C–S bending vibration of the sulfonic acid groups), which corresponds to the chemical structure of SLS [41]. The stretching vibration of the benzene ring and S=O from the sulfonic acid groups are observed at 1596 and 1195 cm^−1^, respectively [42]. The band at 1118 cm^−1^ could be a sign of C-H bond because of syringyl lignin [43]. The broad peak at 1046 cm^−1^ represents the S=O stretching vibration of sulfonate groups [44], which overlaps with the O-H stretching vibration in the guaiacyl lignin. SLS-loaded CaCO_3_ particles could also be indirectly inferred from the broad spectrum between the range of 3390–3450 and 2940–2990 cm^−1^ because of the stretching vibration of O-H bonds and C-H bonds, respectively [45]. These bands are assigned to the alcoholic and phenolic hydroxyl groups and methylene and methyl groups, respectively, which are characteristic for SLS and could be clearly seen in the spectra for the loaded CaCO_3_ particles. The bands in the range of 670–2050 cm^−1^ are from the bending vibrations of the carbonate ions. These overlap with the C-H, S=O, and C-S stretching, respectively, which are related to the presence of SLS.

### 4.2. Corrosion Inhibition Performance of CaCO_3_ Containing-Epoxy Coating

#### 4.2.1. Open Circuit Potential (OCP)

**Figure 7:**
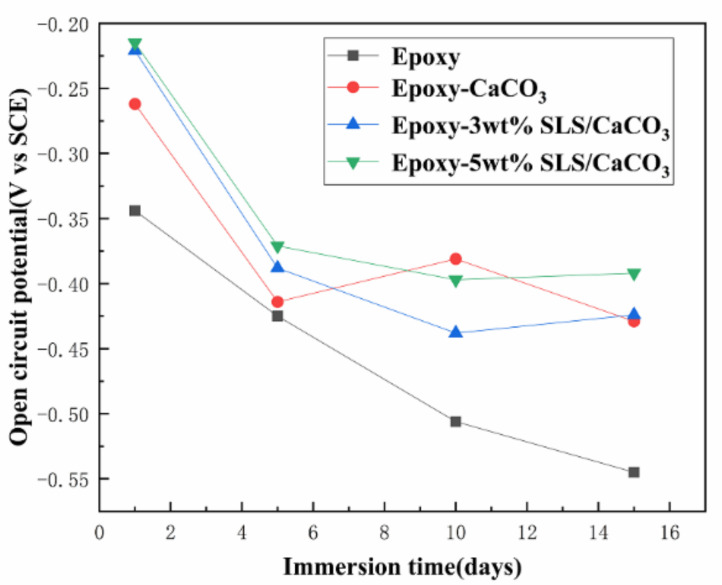
Evolution of open-circuit potential (OCP) of all coating specimens with the immersion time in simulated concrete pore (SCP) solution.

Figure 7 displays the open circuit potentials (OCP) of all specimens in SCP solution. The SLS/CaCO_3_ microcontainers-containing epoxy coatings had a higher OCP value than blank epoxy coating at the initial stage of the immersion. After the prolonging immersion of the coating system, all specimens presented the gradually dropping OCP values. For instance, the OCP value for blank epoxy specimen dropped remarkably from the initial −0.344 to −0.545 V after immersion for 15 days. The open circuit potential of epoxy-CaCO_3_ coating increased slightly on the tenth day, which may be attributed to the corrosion products block penetration path of corrosive medium. Meanwhile, for the epoxy coating modified with SLS/CaCO_3_ microcontainers, the OCP value declined slowly. The OCP value for the epoxy coating containing 3 wt% SLS/CaCO_3_ and 5 wt% SLS/CaCO_3_ presented a decreasing trend (from −0.262 to −0.429 V, from −0.221 to −0.424 V). This confirmed that the inhibitor SLS could release from CaCO_3_ particles and optimized the protective properties of epoxy coating.

#### 4.2.2. Electrochemical Impedance Spectroscopy (EIS)

**Figure 8:**
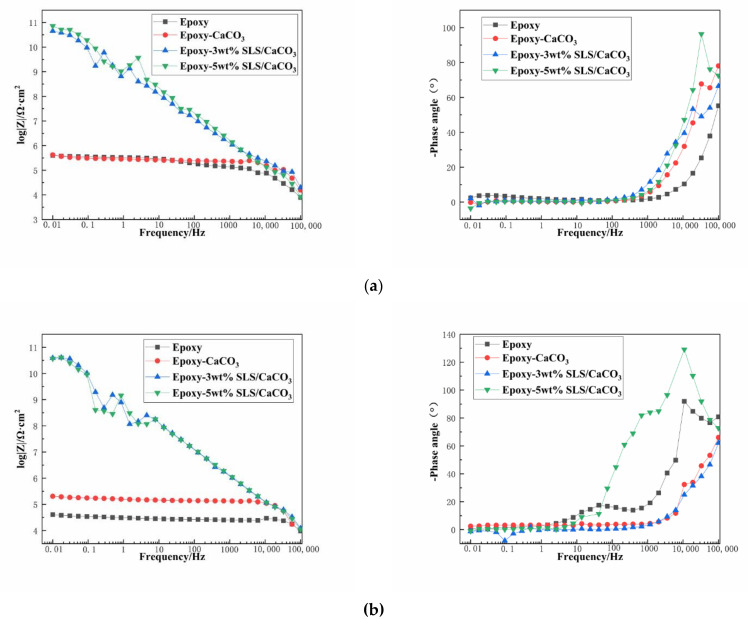
Bode plots for the epoxy coating containing CaCO_3_ microparticle with or without SLS as well as the pure epoxy coating, measured after (**a**) 5 days and (**b**) 15 days exposure in the SCP solutions with 3.5 wt% NaCl.

Figure 8 depicts the EIS diagrams of the blank epoxy coatings and modified epoxy coatings after immersion in the SCP solutions. Figure 8a is the Bode impedance spectra of the samples measured after immersion period of 5 days in the SCP test solutions. The barrier performance of the coating system is usually reflected by the impedance modulus |Z| at the lowest frequency. The impedance value at 0.01 Hz (|Z|_0.01 Hz_) of the coatings containing CaCO_3_ particles with SLS are higher than that of blank epoxy coating during the early stage of immersion period. Especially, the epoxy-5 wt% SLS/CaCO_3_ coating exhibited that the |Z|_0.01 Hz_ value reached 7.22 × 10^10^ Ω·cm^2^, being five order of magnitude higher than that of the blank epoxy coating. The Bode curves of the specimens after 15-day immersion are presented in Figure 8b. For the reference sample, the |Z|_0.01 Hz_ value dropped from 3.99 × 10^5^ to 4.05 × 10^4^ Ω·cm^2^ after 15 days, which was slightly lower than that of the epoxy coating containing CaCO_3_ particles in the absence of SLS (from 4.20 × 10^5^ to 2.04× 10^5^ Ω·cm^2^) after 15 days of immersion in the SCP solution. The |Z|_0.01 Hz_ value decreased from 4.52 × 10^10^ to 3.88 × 10^10^ Ω·cm^2^ for the epoxy-3wt%SLS/CaCO_3_ coating. In addition, the coating modified by the 5 wt% CaCO_3_ particles loaded with SLS exhibited the higher |Z|_0.01 Hz_ values at the early stage, and then, the values presented a slow descending trend, reaching values approximately 3.73 × 10^10^ Ω·cm^2^.

The protective performance of the coating system was illustrated by the Bode phase angle. The peaks appear at the range of 10^−2^–1 Hz (low frequency region), owing to the corrosion activity at interface of the metallic surface and the coating; slight defects or pores on the coatings are reflected by the peak exhibiting within the range of 1 Hz to 10^3^ Hz (intermediate frequency region); the shielding behavior of the coating can be observed by the peak at 10^4^–10^5^ Hz (high frequency region) [46]. In the high frequency region, the coating modified with 5 wt% SLS/CaCO_3_ exhibited pronounced peak under all immersion durations, suggesting that a better anticorrosion property than the other coatings. The phase angle of the epoxy-5wt% SLS/CaCO_3_ coating at high frequency was relatively higher than that of the epoxy coating with 3 wt% SLS/CaCO_3_ particles. For the blank coating, the peak appeared at the intermediate frequency region after 15 days of immersion, revealing the existence of tiny defect on the coating.

**Figure 9:**
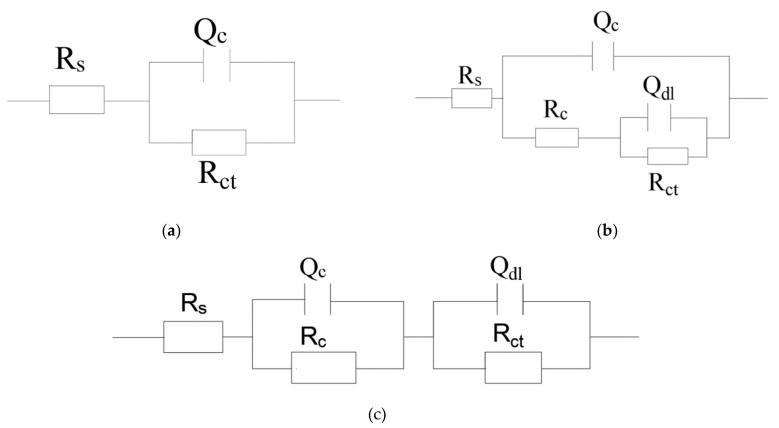
Equivalent circuit: (**a**) one-time constant model and (**b**), (**c**) two-time constants model.

The presence of one-time constant corresponding to the coatings at the initial stage of the soaking were fitted by equivalent electrical circuit (Figure 9a) to model the EIS spectra. The impedance spectra of the coating at the later period of immersion revealed two-time constants: the inhibition properties of the coating are reflected at lower frequency, while the other at higher frequency indicates the corrosion phenomena appearing on the metal-coating interface [47]. 

Those EIS data were modeled applying equivalent circuit, as shown in Figure 9b,c. The corrosion medium diffuses into the interface between coating and metal through micropore on the coating. The equivalent circuit of this situation corresponds to Figure 9b. If the corrosive media evenly penetrates into the coating system. The corresponding equivalent circuit is shown in Figure 9c. The related parameters for the corresponding coating fitted by ZSimpWin software are provided in Table 2. The proposed equivalent circuit consists of the solution resistance (R_s_), capacitance of the coating (Q_c_), resistance of the coating (R_c_), charge transfer resistance (R_ct_), and double-layer capacitance (Q_dl_) [16]. The value of n close to ~ 1 represents that the steel-electrode can be approximated by the pure capacitance. Otherwise, the n value is about zero, which indicates that the steel electrode can be regarded approximately as a pure resistance [48].

The anticorrosive performance of the coating can be evaluated by the R_c_ value. The higher value of R_c_ indicates better corrosion protection of the coating. Specific changes exist in the R_c_ values with the increase in soaking time. For all the coatings, the values of R_c_ declined because of the invasion of the electrolyte and the accumulation of chloride ions between the coating and the steel surface. Thanks to the CaCO_3_ particles as fillers in the coating, the epoxy-CaCO_3_ coating R_c_ value (9.53 × 10^4^ Ω·cm^2^) was relatively higher than that of pure epoxy coating. The coatings modified by 3 wt% and 5 wt% SLS/CaCO_3_ micro-containers had higher values of R_c_ (5.16 × 10^10^ and 4.54 × 10^10^Ω·cm^2^), while the R_c_ value of blank epoxy coating was 1.96 × 10^4^ Ω·cm^2^ over the 15 days of testing period. Observation demonstrates that the tortuous penetration path of the aggressive medium thorough the coating can be increased by these micro-containers. This trend evidenced that the addition of SLS/CaCO_3_ microparticles significantly enhanced the corrosion resistance of the epoxy coating.

The evolution of the R_ct_ value is related to the corrosion protection of the coating [42]. The R_ct_ value of epoxy-SLS/CaCO_3_ coating was much higher than that of blank epoxy coating after immersion for 15 days, implying that its better corrosion-protective ability compared to the pure epoxy specimen.

The variation of the Q_dl_ reflects the delamination of the interface of metal and coating. Therefore, the lower Q_dl_ represents that the excellent barrier effect of coating [49]. The epoxy coating modified with CaCO_3_ particles exhibited lower Q_dl_ value than neat epoxy coating. Thus, the metal-coating interface reaches stabilization because of the presence of CaCO_3_ particles.

Q_c_ means the water absorption of coating. The penetration of corrosive electrolytes accompanied by an increase in Q_c_. The Q_c_ values for all the coatings are stable during 15 days of immersion, revealing all the coatings still have barrier ability.

The Q_c_ and Q_dl_ represents the prefactor of a constant phase element (CPE)to replace the pure capacitance because of the heterogeneities of surface. The pure capacitance can be calculated as follows [50]

(4) $$C_{x}={\left(Q_{x\;}R_{x}{}^{1-n_{x}}\right)}^{1/n_{x}},$$

where *C_x_*, *Q_x_*, and *n_x_* represents the capacitance, the constant-phase element, and the empirical exponent, respectively, R_x_ is either the pore resistance of the coating (R_c_) or charge transfer resistance (R_ct_). 

**Figure 10:**
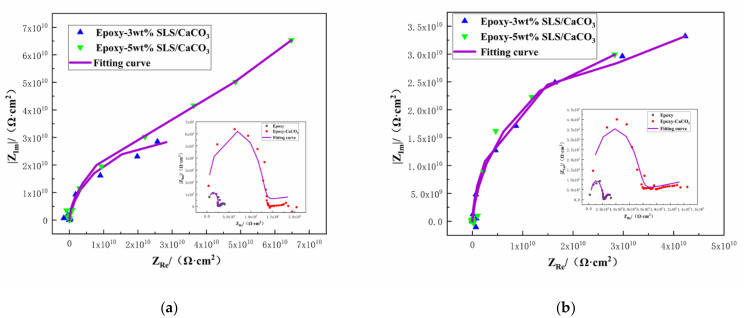
Nyquist plots for Q235 steel covered by epoxy coatings containing CaCO_3_ microparticles with or without SLS as well as the pure epoxy coating after exposure to the SCP solution (**a**) 5 days and (**b**)15 days.

The protective property of the coatings can be acquired from the Nyquist plots [51]. The Nyquist plots of the coating system during 15 days immersion in SCP solutions are depicted in Figure 10. Larger the diameter of the capacitive loop means better the inhibition action. As shown in Figure 10, the diameter of the capacitive loop of all the coated steels decreases slightly after longer exposition time. However, the reference coating exhibited lower capacitive loop than those modified with the SLS/CaCO_3_ micro-containers, demonstrating superior inhibition performance of the epoxy-SLS/CaCO_3_ coating.

#### 4.2.3. Potentiodynamic Polarization Measurement

**Figure 11:**
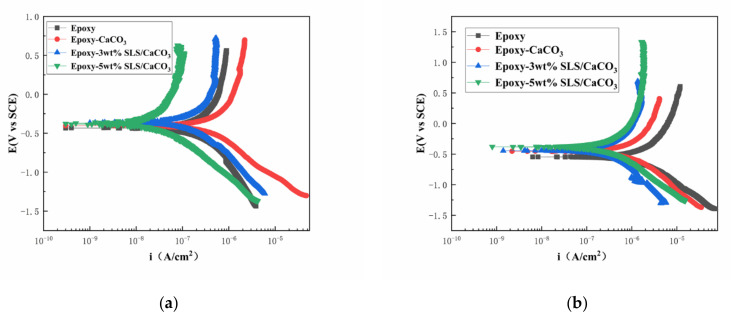
Potentiodynamic polarization curves for the epoxy coating containing CaCO_3_ microparticles with or without SLS as well as the pure epoxy coating, measured after (**a**) 5 days and (**b**) 15 days exposure in the SCP solutions with 3.5 wt% NaCl.

The polarization curves of the coated steel after 5 and 15 days of immersion in the SCP solutions are illustrated in Figure 11. Table 3 reports the kinetic parameters obtained from polarization curves, including the corrosion potential (E_corr_), the corrosion current density (*i_corr_*), and the inhibition efficiency (IE%). The inhibition efficiency was defined using Equation (5)

(5) $$\mathrm{IE}\%=\frac{i_{corr}\left(\mathrm{blank}\;\mathrm{epoxy}\;\mathrm{coating}\right)-i_{corr}\left(\mathrm{modified}\;\mathrm{epoxy}\;\mathrm{coating}\right)}{i_{corr}\left(\mathrm{blank}\;\mathrm{epoxy}\;\mathrm{coating}\right)}.$$

As shown in Figure 11, the curves of the coatings modified with SLS/CaCO_3_ micro-containers shifted toward more positive potentials and exhibited a decreased i_corr_. This suggests that the addition of SLS/CaCO_3_ particles can further enhance the inhibition performance of the epoxy coating. In Table 3, in comparison to the pure epoxy coating, the E_corr_ of 3 wt% SLS/CaCO_3_ epoxy coating and 5 wt% SLS/CaCO_3_ epoxy coating moved to more positive position of 0.042 and 0.059 V, respectively, after 5 days of immersion. Moreover, it was noticed that the IE% of 5 wt% SLS/CaCO_3_ epoxy coating reached 90.83%. After immersion period of 15 days, the *i_corr_* values of the coating system showed increasing trend. The *i_corr_* values obtained for epoxy coating, epoxy-CaCO_3_ coating, epoxy-3 wt% SLS/CaCO_3_ coating and epoxy-5wt%SLS/CaCO_3_ coating were 1.12 × 10^−6^, 3.73 × 10^−7^, 1.90 × 10^−7^, and 1.72 × 10^−7^ A·cm^2^ after 15 days of immersion, respectively. Surprisingly, the i_corr_ value of modified coating was one orders of magnitude higher than that blank epoxy coating. The E_corr_ value for the epoxy-5wt%SLS/CaCO_3_ coating remained stable (from −0.371 to −0.378 V), while the E_corr_ value of the neat epoxy coating decreased in the range from −0.430 to −0.556 V. The IE% values of epoxy coating containing 3wt% SLS/CaCO_3_ and 5wt%SLS/CaCO_3_ were 83.02 and 84.64, respectively. Therefore, it suggests that SLS releases from CaCO_3_ micro-containers.

#### 4.2.4. Physical Properties of Epoxy Composite Coatings

**Figure 12:**
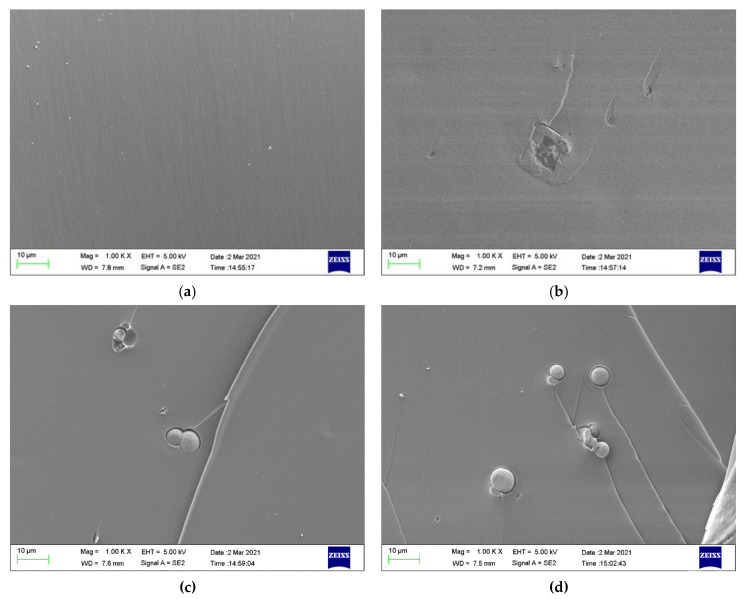
Cross-section SEM of (**a**) epoxy coating (**b**) epoxy-CaCO_3_ coating, (**c**) epoxy-3 wt%SLS/CaCO_3_ coating, and (**d**) epoxy-5 wt%SLS/CaCO_3_ coating after exposure to the SCP solutions for 48 h.

**Figure 13:**
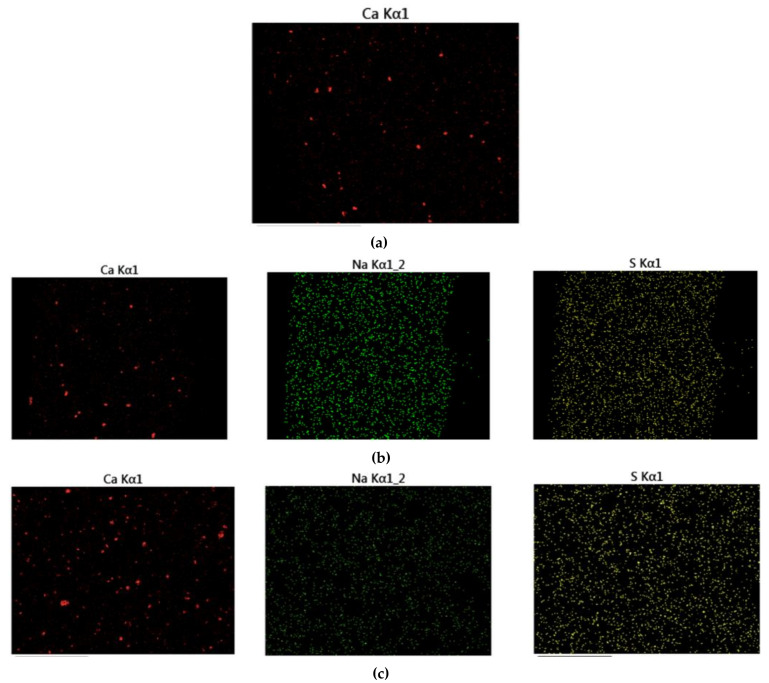
The EDX map of (**a**) epoxy-CaCO_3_ coating, (**b**) epoxy-3 wt%SLS/CaCO_3_ coating, and (**c**) epoxy-5 wt%SLS/CaCO_3_ coating.

Figure 12 displays the fracture cross-sectional morphologies of different epoxy coatings after being immersed in the SCP solutions. For pure epoxy coating, the cross-section presented the relatively smooth surface. (Figure 12a). In Figure 12b–d, the CaCO_3_ particles were blended into the epoxy coating. After the addition of CaCO_3_ particles, the fracture surface of the modified coatings manifested rough surface. CaCO_3_ micro-container exhibited the better compatibility with the blank epoxy coating. The EDX elemental mapping of the epoxy-CaCO_3_, epoxy-3 wt% SLS/CaCO_3_, and epoxy-5 wt% SLS/CaCO_3_ specimens is depicted in Figure 13. Ca, Na, and S elements were dispersed homogeneously, indicating that the SLS/CaCO_3_ particles were distributed evenly in the epoxy matrix.

**Figure 14:**
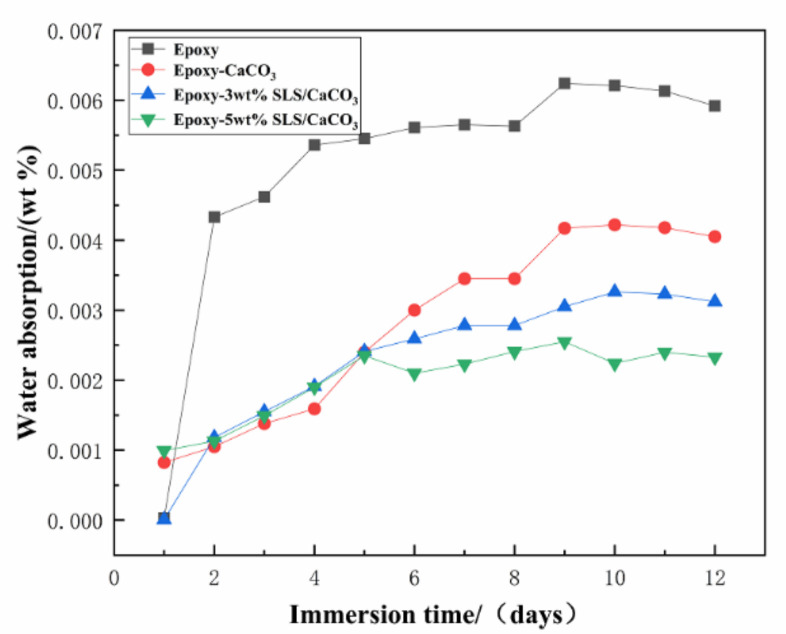
Water absorption of various coatings over immersion time.

Permeation of water and anti-corrosive behavior of the coating can be measured by the water absorption test. The water absorptions of all the coatings are displayed in Figure 14. With increasing immersion time, a rapid rise in the water absorption values reveals the diffusion of water into the coating defects. Then, the coating water absorption was at a stable value, indicating that the liquid in the coating defects reached saturation state. After 12 days of immersion, the water absorption of the pure epoxy coating, epoxy-CaCO_3_ coating, epoxy-3 wt% SLS/CaCO_3_ coating, and epoxy-5 wt% SLS/CaCO_3_ coating were 0.592%, 0.405%, 0.312% and 0.233%, respectively. The modified coating possesses lower water absorption value and can be ascribed to the calcium carbonate filler reducing the porosity of the coating.

**Figure 15:**
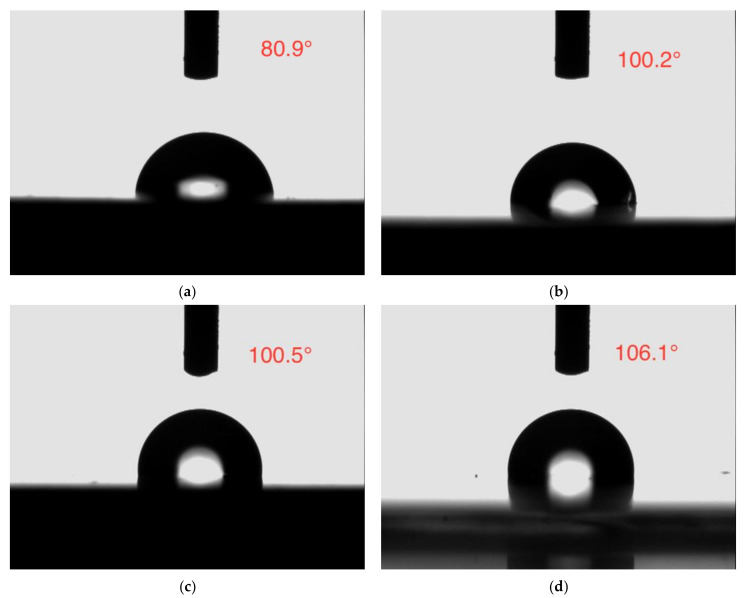
Contact angle images of (**a**) epoxy coating, (**b**) epoxy-CaCO_3_ coating, (**c**) epoxy-3 wt%SLS/CaCO_3_ coating, and (**d**) epoxy-5 wt%SLS/CaCO_3_ coating.

The contact angle analysis is a frequently used method to confirm the wettability of the coating, which is related to the barrier protection effect of the coating [52].The water contact angle photographs of all the coatings are depicted in Figure 15. The neat epoxy coating displayed a lower contact angle of 80.9°, while the contact angle of the epoxy coating incorporated with CaCO_3_, 3 wt% SLS/CaCO_3_, and 5 wt% SLS/CaCO_3_ were 100.2°, 100.5°, 106.1°, respectively, symbolizing the improved hydrophobicity of the modified coating specimens.

The results of the coating system after adhesion measurements are showed in Table 4. The adhesion level for pure epoxy coating, epoxy-CaCO_3_ coating, epoxy-3 wt% SLS/CaCO_3_ coating, and epoxy-5 wt% SLS/CaCO_3_ coating were 1, 0, 0, and 0, respectively. These observations illustrate that the modification by SLS/CaCO_3_ micro-containers can effectively enhance the adhesion strength of the pure epoxy resin. It can be inferred that the epoxy-SLS/CaCO_3_ coating possesses better adhesive property because the moderate amount of SLS/CaCO_3_ micro-container was added in neat epoxy coating. Furthermore, CaCO_3_ particles as filler will generate physical or chemical bond with the metal substrate, so as to ensure that the coating can adhere to the metal surface well [53]. 

The coating hardness of the pure epoxy coatings was similar to those coatings modified with the CaCO_3_ micro-containers, as shown in Table 5. All the coating displayed better hardness performance.

### 4.3. Mechanism of Inhibition Protection

Importantly, 3CaO·SiO_2_ and 2CaO·SiO_2_ are the main components of Portland concrete cement. A large amount of calcium hydroxide is generated after hydration reaction, which will lead to the concrete pore solution with high alkalinity [54]. In the case of high pH value, a passive film (Fe(OH)_3_) precipitates on the carbon surface and hinders the invasion of corrosive medium. Under severe environment, corrosive media such as aggressive chloride ion and carbon dioxide will cause the decline in the pH value of the concrete and the destruction of protective film on the metallic substrate [55]. At this time, it is necessary to apply some practical methods on corrosion control of the reinforced concrete. Under acidic condition, an active release of SLS may contribute to the dissolution of CaCO_3_ micro-containers in response to the change of pH. Increasing pH of the SCP solution due to continuous release of SLS results in the formation of passive film on carbon surface. Furthermore, SLS contains hydrophobic skeleton and hydrophilic groups (hydroxyl groups and sulfonic groups). The interaction between the SLS with sulfonic groups and positively charged metal surfaces is responsible for chemisorption. Moreover, the SLS is absorbed on the carbon surface due to the formation of coordinative bonds between the metal ions and the O element in phenolic hydroxyl group of SLS. The optimum addition of 5 wt% SLS/CaCO_3_ not only improves the self-healing capability of the pure epoxy coating but also avoids the consumption of corrosion inhibitor when the steel bar is in the passivated state.

## 5. Conclusions

In this study, calcium carbonate was used as pH-sensitive micro-containers for the loading of SLS as corrosion inhibitor, which were confirmed with FTIR, XRD, and SEM. Under the presence of different pH solutions, the response of SLS-loaded CaCO_3_ micro-containers was investigated by UV-vis. The corrosion inhibition effect of incorporating various amount (3 wt% and 5 wt%) of SLS/CaCO_3_ microparticles with the epoxy coating in the SCP solutions was discussed by electrochemical measurements and a series of physical properties tests. The following observations were made:

1. The CaCO_3_ microparticles modified with sodium lignosulfonate had the same oval-shaped morphology as the unmodified one.
2. XRD results showed that the CaCO_3_ particles with or without SLS contain both the vaterite and calcite phases. Presence of sodium lignosulfonate in the CaCO_3_ particles leads to more calcite phase.
3. The SLS loaded into the CaCO_3_ micro-containers can be released under different pH conditions, with the extent of sodium lignosulfonate release in the acidic media being higher.
4. Electrochemical measurements demonstrated the beneficial role of the SLS/CaCO_3_ microparticles on the inhibition performances of the epoxy coating, especially the epoxy-5 wt% SLS/CaCO_3_ coating. These microparticles show better compatibility with the original epoxy coating.
5. The epoxy-SLS/CaCO_3_ coating showed high hydrophobicity and good mechanical strength and exhibited better adhesion on the substrate.

## Figures and Tables

**Table 1:** Time dependent release of sodium lignosulfonate (SLS) from the CaCO_3_ micro-containers under different pH values of the aqueous solutions.

Time/(h)	pH	Absorbance/(a.u.)	Concentration/(mg/L)
24	4	0.107	38.816
	7	0.075	30.395
	10	0.039	20.921
48	4	0.104	38.026
	7	0.058	25.921
	10	0.038	20.658
72	4	0.094	35.395
	7	0.058	25.921
	10	0.049	23.553

**Table 2:** Fitting electrochemical data of the applied coatings in the simulated concrete pore (SCP) solutions.

Specimens	Time (Day)	R_s_ (Ω·cm^2^)	Q_c_ (Ω^−1^·cm^−2^·s^n^)	n	R_c_ (Ω·cm^2^)	Q_dl_ (Ω^−1^·cm^−2^·s^n^)	n	R_ct_ (Ω·cm^2^)	χ^2^	Equivalent Circuit
Epoxy	5	2799	3.33 × 10^−10^	9.3 × 10^−1^	2.76 × 10^4^	–	–	–	0.02710	R(QR)
	15	2266	3.11 × 10^−10^	1	1.96 × 10^4^	7.65 × 10^−5^	4.48 × 10^−1^	1.63 × 10^4^	0.01538	R(QR)(QR)
Epoxy-CaCO_3_	5	0.04873	4.54 × 10^−11^	1	1.59 × 10^5^	–	–	–	0.02607	R(QR)
	15	132	7.10 × 10^−11^	2.45 × 10^−1^	9.53 × 10^4^	1.01 × 10^−5^	1	7.16 × 10^4^	0.00910	R(QR)(QR)
Epoxy-3 wt% SLS/CaCO_3_	5	750	1.22 × 10^−10^	9.45 × 10^−1^	5.20 × 10^10^	–	–	–	0.04723	R (QR)
	15	2930	1.09 × 10^−10^	1	5.16 × 10^10^	1.40 × 10^−5^	6.12 × 10^−1^	1.30 × 10^10^	0.02086	R(Q(R(QR)))
Epoxy-5 wt% SLS/CaCO_3_	5	3166	9.03 × 10^−11^	1	9.55 × 10^10^	–	–	–	0.02707	R (QR)
	15	1662	1.40 × 10^−10^	9.79 × 10^−1^	4.54 × 10^10^	7.94 × 10^−7^	9.99 × 10^−1^	4.36 × 10^10^	0.03265	R(Q(R(QR)))

**Table 3:** Polarization parameters of the applied coatings in the SCP solutions.

Specimens	Time (day)	E_corr_ (V)	*i_corr_* (A·cm^2^)	*IE* (%)
Epoxy	5	−0.430	1.92 × 10^−7^	−
	15	−0.556	1.12 × 10^−6^	−
Epoxy-CaCO_3_	5	−0.401	2.44 × 10^−7^	−
	15	−0.452	3.73 × 10^−7^	66.69
Epoxy-3wt%SLS/CaCO_3_	5	−0.388	9.05 × 10^−8^	52.86
	15	−0.445	1.90 × 10^−7^	83.02
Epoxy-5wt%SLS/CaCO_3_	5	−0.371	1.76 × 10^−8^	90.83
	15	−0.378	1.72 × 10^−7^	84.64

**Table 4:** Adhesion level of different coatings.

Specimens	Adhesion Level
Epoxy	1
Epoxy-CaCO_3_	0
Epoxy-3 wt%SLS/CaCO_3_	0
Epoxy-5 wt%SLS/CaCO_3_	0

**Table 5:** The pencil hardness of all the coatings.

Specimens	Scratch	Gauge
Epoxy	6H	4H
Epoxy-CaCO_3_	6H	4H
Epoxy-3 wt%SLS/CaCO_3_	6H	4H
Epoxy-5 wt%SLS/CaCO_3_	6H	4H

## Data Availability

Data is contained within the article.

## Abbreviation

SLS	Sodium lignosulfonate
CaCO_3_	Calcium carbonate
SCP solution	Simulated concrete pore solution
SLS/CaCO_3_	Calcium carbonate microparticles loaded with SLS
SEM	Scanning electron microscope
UV-vis	UV–VIS spectrophotometer
XRD	X-ray diffraction
ATR-FTIR	attenuated total reflection-Fourier-transform infrared spectroscopy
OCP	Open-circuit potential
EIS	Electrochemical impedance spectroscopy
Epoxy-3 wt%SLS/CaCO_3_ coating	The epoxy coating modified by the 3 wt% calcium carbonate particles loaded with sodium lignosulfonate
Epoxy-5 wt%SLS/CaCO_3_ coating	The epoxy coating modified by the 5 wt% calcium carbonate particles loaded with sodium lignosulfonate
